# Sodium citrate versus sodium bicarbonate for metabolic acidosis in patients with chronic kidney disease: A randomized controlled trial

**DOI:** 10.1097/MD.0000000000037475

**Published:** 2024-03-08

**Authors:** Bogdan Marian Sorohan, Bogdan Obrișcă, Roxana Jurubiță, Gabriela Lupușoru, Camelia Achim, Andreea Andronesi, Georgiana Frățilă, Andreea Berechet, Georgia Micu, Gener Ismail

**Affiliations:** a “Carol Davila” University of Medicine and Pharmacy, Department of Nephrology, Bucharest, Romania; b Fundeni Clinical Institute, Department of Kidney Transplantation, Bucharest, Romania; c Fundeni Clinical Institute, Department of Nephrology, Bucharest, Romania.

**Keywords:** adverse events, bicarbonate, chronic kidney disease, citrate, decline, GFR, metabolic acidosis, progression, randomized, sodium, trial

## Abstract

**Background:**

Metabolic acidosis (MA) is frequently associated with chronic kidney disease (CKD) progression. Our aim was to compare the effect of oral sodium citrate (SC) with that of oral sodium bicarbonate (SB) on renal function and serum bicarbonate correction, as well as to evaluate their safety profile in patients with MA of CKD.

**Methods:**

We conducted a prospective, single-center, randomized 1:1, parallel, controlled, unblinded clinical trial of 124 patients with MA and CKD stages 3b and 4. The primary outcome was the mean change in estimated glomerular filtration rate (eGFR). The secondary outcomes were mean change in serum bicarbonate level, eGFR decrease by 30%, eGFR decrease by 50%, dialysis, death or prolonged hospitalization, and a combined endpoint.

**Results:**

No significant difference was found between the groups in terms of mean eGFR change [adjusted mean difference = −0.99 mL/min/1.73 m^2^ (95% CI: −2.51 to 0.93, *P* = .20)]. We observed a mean serum bicarbonate change of 6.15 mmol/L [(95% CI: 5.55–6.74), *P* < .001] in the SC group and of 6.19 mmol/L [(95% CI: 5.54–6.83), *P* < .001] in the SB group, but no significant difference between the 2 groups [adjusted mean difference = 0.31 mmol/L (−0.22 to 0.85), *P* = .25]. Cox proportional hazard analysis showed similar risks regarding eGFR decrease by 30% (*P* = .77), eGFR decrease by 50% (*P* = .50), dialysis (*P* = .85), death or prolonged hospitalization (*P* = .29), and combined endpoint (*P* = .57). Study drug discontinuation due to adverse events was significantly more common in the SB group (17.7% vs 4.8%, *P* = .02).

**Conclusions:**

SC and SB have a similar effect on kidney function decline, both improve serum bicarbonate level, but SB is associated with higher rates of medication discontinuation due to adverse events.

## 1. Introduction

The kidney plays a central role in maintaining the acid-base balance by excreting acids, generating and reabsorbing bicarbonate.^[1]^ Patients with chronic kidney disease (CKD) often develop metabolic acidosis(MA), which is defined as a serum bicarbonate level < 22 mmol/L.^[2]^ The prevalence of MA in CKD increases with a decline in kidney function; thus, it can reach up to ~40% in stage 4 CKD.^[3]^ Chronic MA is associated with several complications including cardiovascular events, insulin resistance, endothelial dysfunction, sarcopenia, bone mineral disorders, vascular calcification, and inflammation.^[4]^ Moreover, MA has been shown to be an important risk factor for the progression of CKD. Recent data suggest that acidosis, even in the early stages of CKD, leads to progressive kidney dysfunction.^[5]^

Data from a recent meta-analysis of clinical trials that compared the use of oral alkali supplementation or reduction in dietary acid intake with no treatment, standard of care, or placebo in patients with CKD stage 3 to 5 and MA, found that alkali therapy significantly increased serum bicarbonate, reduced the rate of kidney function decline, reduced proteinuria, and the progression to end-stage renal disease.^[6]^ The positive findings from clinical trials have laid the foundation for treatment recommendations for MA in CKD.^[7]^

Although there is evidence to support alkali therapy for preserving kidney function in patients with MA and CKD, to our knowledge, no clinical trial has compared the efficacy and safety of sodium citrate with sodium bicarbonate in these patients. Thus, the aim of the Sodium Citrate versus Sodium Bicarbonate for metabolic acidosis in patients with chronic kidney disease study was to compare the effect of oral sodium citrate with that of oral sodium bicarbonate on kidney function and serum bicarbonate correction, as well as to compare the safety profile of these alkali therapies in patients with MA of CKD. The hypothesis of this study was that sodium citrate and sodium bicarbonate might have the same effect on slowing the progression of CKD and correcting acidosis, but sodium citrate might be better tolerated.

## 2. Materials and methods

### 2.1. Study design and participants

**Figure 1.**
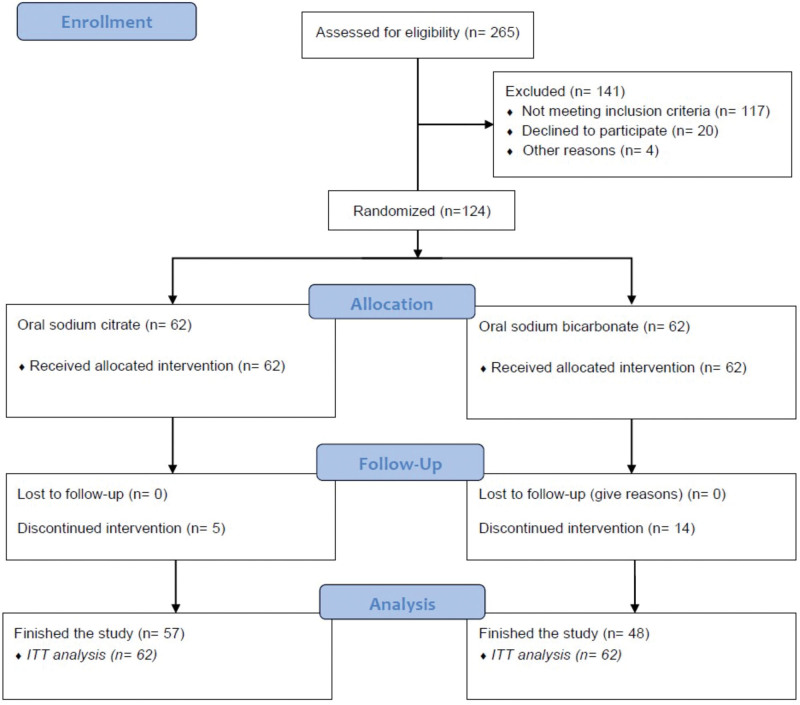
Study flow diagram.

This study was a prospective, single-center, randomized 1:1, parallel, controlled, and unblinded clinical trial. The trial was approved by the Ethics Committee of Fundeni Clinical Institute (date of approval September 20, 2021, no. 59531) and registered in the ISRCTN registry (registration date: September 22, 2021, no. 16429332, https://doi.org/10.1186/ISRCTN16429332). All patients with CKD stage G3b-G4 and MA evaluated in the Nephrology Department of Fundeni Clinical Institute between October 2021 and October 2022, who met the study inclusion criteria, were enrolled (Fig. 1). Inclusion criteria were: age > 18 years, estimated GFR (eGFR) between 45 and 15 mL/min/1.73 m^2^, serum bicarbonate between 10 and 22 mmol/L on two separate measurements, ability to follow the study treatment regimen, and a wash-out period of 1 month if previous alkali therapy was used (sodium bicarbonate, sodium citrate, potassium citrate, baking soda, etc.). Exclusion criteria included hypokalemia < 3 mmol/L, uncontrolled high blood pressure (>150/90 mm Hg under treatment with more than 3 different classes of antihypertensive drugs, including diuretics), heart failure with active class III or IV New York Heart Association, known left ventricular ejection fraction ≤ 30% or hospital admission for heart failure within the past 3 months, hypervolemia of any cause (nephrotic syndrome, liver, or heart failure) considered unsafe, active hepatic disease, chronic gastrointestinal disorder (treatment adherence unreliable), active malignancy, pregnancy, patients taking amiloride or sevelamer, and patients who refused to sign the informed consent.

### 2.2. Intervention and measurements

Participants were randomly assigned to sodium citrate or sodium bicarbonate groups (1:1) based on a computer random number generator. Patients in the sodium citrate group received sodium citrate powder 1997 mg/d if serum bicarbonate was 19 to 22 mmol/L or 1997 mg twice daily if serum bicarbonate level was < 18 mmol/L. If serum bicarbonate remained below the target value at the next follow-up, the dose was increased to a maximum of 7988 mg (divided into 3 or 4 intakes per day). Patients from the sodium bicarbonate group received 1 tablet of 600 mg sodium bicarbonate/day if serum bicarbonate was 19 to 22 mmol/L or 600 mg twice daily if serum bicarbonate was under 18 mmol/L. If serum bicarbonate persisted below the target value at the next follow-up, the dose was increased by 1 tablet to a maximum dose of 3600 mg. Patients treated with sodium citrate were instructed to administer the medication at regular intervals by dissolving each sachet (dose) in 200 mL water. They were also instructed on the storage conditions of the sodium citrate sachets (in a cardboard box at room temperature). Patients treated with sodium bicarbonate tablets were instructed to administer the medication at regular intervals after the main meals with a glass of water in 1 or 2 doses, as needed. If the serum bicarbonate level reached 27 to 28 mmol/L, the doses of sodium citrate and sodium bicarbonate were reduced by 50%; if the level was ≥ 29 mmol/L, the medication was discontinued. The doses were changed based on data obtained on the same day.

The study visits were performed monthly, and clinical-biological evaluations were performed at each visit. Clinical assessment consisted of blood pressure measurement, weighing, and adverse event status. Laboratory analyses were performed locally according to the standards of care. The analyzed parameters included complete blood count, serum albumin, urea, calcium, phosphorus, sodium, potassium, chloride, acid-base parameters (pH, bicarbonate, and pCO2), and urinary parameters (proteinuria and potassium). eGFR was evaluated using the Chronic Kidney Disease Epidemiology Collaboration 2021 formula based on creatinine levels.

### 2.3. Outcomes

The primary outcome was the mean change in eGFR after 12 months of treatment. The secondary outcomes were mean change in serum bicarbonate level, eGFR decrease by 30%, eGFR decrease by 50%, kidney failure and dialysis initiation, death or prolonged hospitalization, a combined endpoint (eGFR decrease by 30% or eGFR decrease by 50% or dialysis initiation or death or prolonged hospitalization), and drug safety profile.

### 2.4. Statistical analysis

Continuous parametric variables were presented as mean ± standard deviation (SD), continuous non-parametric variables as median with interquartile range (IQR), and categorical variables as percentages. The following tests were used to compare differences at baseline, depending on the type of variable: Student *t* test for continuous parametric variables, Mann–Whitney *U* test for continuous non-parametric variables, chi-square test, and Fisher’s exact test as appropriate for categorical variables. The mean change from the baseline for continuous variables in each treatment group was obtained using a paired sample *t* test. The mean change in eGFR and serum bicarbonate levels between the 2 treatment groups was expressed as an adjusted mean difference with 95% confidence interval (CI). This was performed using analysis of covariance, considering baseline parameters as covariates. Time-dependent secondary outcome analysis was performed using Cox regression analysis. A forest plot showing the hazard with a 95% CI for each endpoint was constructed. The eGFR slope for each treatment group was analyzed during the treatment period at 3, 6, 9, and 12 months. Statistical analyses were performed using the SPSS version 26 (SPSS Inc. Software, Chicago, IL). Figures were created using GraphPad Prism version 10.0.0 (1992–2023 GraphPad Software, LLC, San Diego, CA) and Biorender. A *P* value < .05 was considered statistically significant.

## 3. Results

### 3.1. Baseline characteristics

In total, 265 patients were assessed for eligibility. Among them, 124 fulfilled the eligibility criteria and were randomized 1:1 to receive either oral sodium citrate or sodium bicarbonate. In the sodium citrate group, 57 of 62 patients completed the study, 3 patients discontinued the medication due to adverse events, and 2 patients stopped the medication on their own initiative. In the sodium bicarbonate group, 48 of 62 patients completed the study, 11 withdrew the medication due to adverse events, 2 patients stopped the medication on their own initiative, and 1 patient died (Fig. 1). All the patients were included in the intention-to-treat analysis.

Baseline characteristics of the study participants are presented in Table 1. The study cohort had a mean age of 57.66 ± 10.20 years and 56.5% were males. At baseline, 42.7% of patients had diabetes, 96% had high blood pressure, and approximately half of them received antihypertensive treatment with angiotensin conversion enzyme inhibitors (ACEi) or angiotensin receptor blockers (ARBs). The mean eGFR at baseline was 24.31 ± 7.95 mL/min/1.73 m^2^ and mean serum bicarbonate at baseline was 17.35 ± 1.85 mmol/L. No statistically significant differences were noted between the sodium citrate and sodium bicarbonate groups at baseline, except for anti-hypertensive treatment with ACEi or ARBs, which were more frequently observed in patients treated with sodium citrate (64.5% vs 40.3%, *P* = .007).

**Table 1:** Baseline characteristics of the study groups.

Variables	Entire cohort (N = 124)	Sodium citrate (N = 62)	Sodium bicarbonate (N = 62)	*P* value
Age (mean ± SD, yr)	57.66 ± 10.20	57.95 ± 10.22	57.37 ± 10.25	.75
Gender (%)				
Male	70 (56.5)	35 (56.5)	35 (56.5)	1
Female	54 (43.5)	27 (43.5)	37 (43.5)	
CKD cause (%)				
DKD	53 (42.7)	27 (43.5)	26 (41.3)	.93
Other	71 (57.3)	35 (56.5)	37 (58.7)	
HTN (%)	119 (96.0)	61 (98.4)	58 (93.5)	.35
eGFR at baseline (mean ± SD, mL/min)	24.31 ± 7.95	24.93 ± 6.70	23.68 ± 9.05	.38
BMI at baseline (mean ± SD, kg/m^2^)	27.82 ± 3.55	27.55 ± 3.70	28.09 ± 3.40	.39
ACEi or ARB (%)	65 (52.4)	40 (64.5)	25 (40.3)	.007
Number of anti-HTN drugs at baseline [median (IQR)]	2.5 (2–3)	3 (2–3)	2.5 (2–3)	.44
Proteinuria at baseline [median (IQR), g/24 h]	1.50 (0.80–2.77)	1.42 (0.70–2.72)	1.50 (0.90–3.03)	.27
Serum HCO3^-^ at baseline (mean, mmol/L)	17.35 ± 1.85	17.53 ± 2.05	17.16 ± 1.62	.26
Serum K^+^ at baseline (mean, mmol/L)	4.94 ± 0.39	4.88 ± 0.44	5.00 ± 0.34	.10
Serum Na^+^ at baseline (mean, mmol/L)	138.57 ± 2.10	138.44 ± 2.61	138.71 ± 1.41	.45

ACE = angiotensin conversion enzyme inhibitor, ARB = angiotensin receptor blocker, BMI = body mass index, CKD = chronic kidney disease, DKD = diabetic kidney disease, eGFR = estimated glomerular filtration rate, HCO3 = bicarbonate, HTN = hypertension, IQR = interquartile range, K = potassium, N = number, Na = sodium, NDKD = non-diabetic kidney disease, sCr = serum creatinine, SD = standard deviation.

### 3.2. Primary outcome analysis

The primary outcome analysis showed no difference in the mean change of eGFR between the 2 groups after 12 months of treatment. In the sodium citrate group mean eGFR change per year was −1.58 mL/min/1.73 m^2^ (95% CI: −4.24 to 1.10). In the sodium bicarbonate group mean eGFR change per year was −1.33 mL/min/1.73 m^2^ (95% CI: −4.63 to 2.20). Group comparison showed an adjusted mean difference in eGFR of −0.99 mL/min/1.73 m^2^ (95% CI: −2.51 to 0.93, *P* = .20) (Table 2).

**Table 2:** Intention-to-treat analysis regarding changes from baseline (Δ changes) to 12 months of treatment between the 2 groups.

	Sodium citrate	Sodium bicarbonate		*P* value
	Mean change from baseline (95% CI)	Mean change from baseline (95% CI)	Adjusted mean difference (95% CI)	
Δ eGFR (mL/min)	−1.58 (−4.24 to 1.10)	−1.33 (−4.63 to 2.20)	−0.99 (−2.51 to 0.93)	.20
Δ BMI (kg/m^2^)	0.34 (−1.66 to 0.98)	−0.06 (−1.92 to 1.80)	− 0.01 (−1.24 to 1.22)	.98
Δ Proteinuria (g/24 h)	−0.10 (−0.45 to 0.25)	−0.03 (−0.94 to 0.88)	− 0.04 (−0.20 to 0.10)	.53
Δ HCO3^-^ (mmol/L)	6.15 (5.55–6.74)	6.19 (5.54–6.83)	0.31 (−0.22 to 0.85)	.25
Δ K^+^ (mmol/L)	−0.29 (−0.46 to −0.11)	−0.20 (−0.49 to −0.20)	−0.02 (−0.20 to 0.16)	.20
Δ Na^+^ (mmol/L)	0.21 (−0.62 to 1.04)	−0.06 (−0.62 to 0.50)	−0.01 (−0.69 to 0.66)	.95

BMI = body mass index, CI = confidence interval, eGFR = estimated glomerular filtration rate, HCO3 = bicarbonate, K = potassium, Na = sodium, Δ = change from baseline

**Figure 2.**
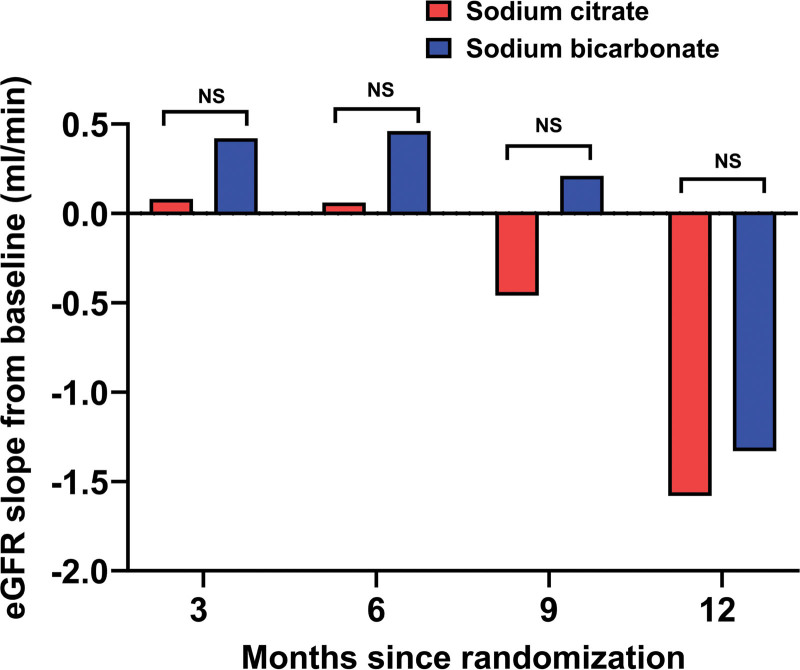
eGFR slope at 3, 6, 9, and 12 months according to treatment group.

The eGFR slope according to randomized treatment assignment is shown in Figure 2. At 3, 6, 9, and 12 months, no statistically significant differences within or between the groups were observed (Table 3).

**Table 3:** eGFR slope according to treatment group and slope difference.

	Sodium citrate			Sodium bicarbonate			Slope difference	
	Mean ± SD	Slope (95% CI)	*P* value	Mean ± SD	Slope (95% CI)	*P* value	(95% CI)	*P* value
Baseline	24.93 ± 6.70			23.68 ± 9.05				
3 mo	25.01 ± 7.80	+0.08 (−2.52 to 2.68)	.95	24.10 ± 9.12	+0.42 (−2.92 to 3.68)	.80	−0.34 (−2.74 to 1.98)	.30
6 mo	24.99 ± 8.31	+0.06 (−2.64 to 2.76)	.96	24.14 ± 9.30	+0.46 (−2.90 to 3.82)	.78	−0.40 (−4.90 to 1.52)	.11
9 mo	24.47 ± 8.31	−0.46 (−3.16 to 2.24)	.73	23.89 ± 9.51	+0.21 (−3.22 to 3.64)	.90	−0.67 (−5.19 to 1.33)	.10
12 mo	23.34 ± 7.98	−1.58 (− 4.23 to 1.05)	.23	22.35 ± 9.51	−1.33 (−4.85 to 2.19)	.45	−0.25 (−4.38 to 2.39)	.20

CI = confidence interval, SD **=** standard deviation.

### 3.3. Secondary outcome analysis

Patients from both groups had a significant mean change in serum bicarbonate after 12 months of treatment [sodium citrate group: 6.15 mmol/L (95% CI: 5.55–6.74), *P* < .001 and sodium bicarbonate group: 6.19 mmol/L (95% CI: 5.54–6.83), *P* < .001]. However, the adjusted mean difference was not statistically significant between the 2 groups [0.31 mmol/L (−0.22 to 0.85), *P* = .25] (Table 2). The mean doses of sodium citrate at baseline, 3, 6, 9, and 12 months were 3204.88 ± 975.93 mg/d, 3394.90 ± 1235.95 mg/d, 3594.60 ± 1551.23 mg/d, 3857.76 ± 1693.82 mg/d, and 3888.01 ± 1746.66 mg/d, respectively. The mean doses of sodium bicarbonate at the aforementioned intervals of time were 1064.51 ± 252.91 mg/d, 1166.03 ± 341.34 mg/d, 1313.20 ± 513.67 mg/d, 1800.00 ± 1009.95 mg/d, and 1927.65 ± 1061.11 mg/d, respectively.

**Figure 3.**
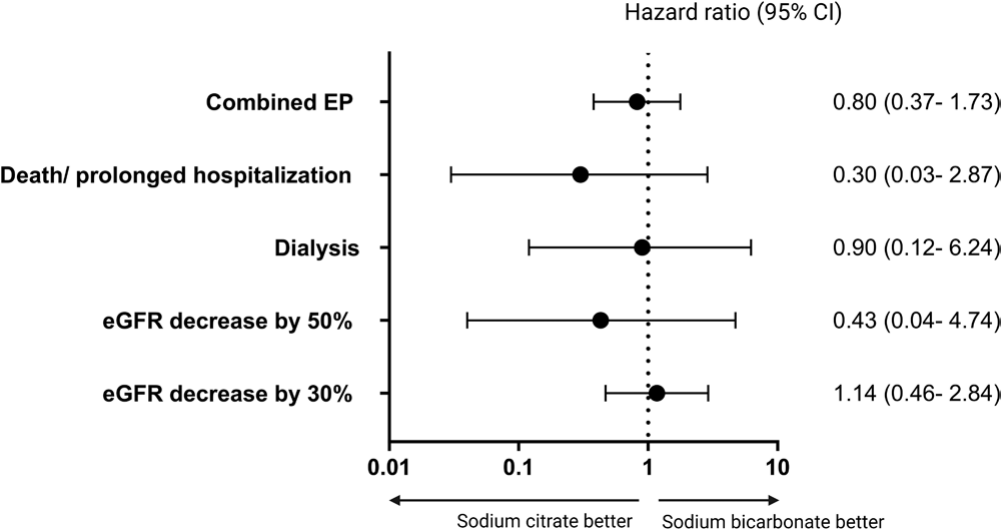
Effect of oral sodium citrate compared to oral sodium bicarbonate on secondary outcomes.

The Cox proportional-hazard analysis showed similar risks in patients treated with sodium citrate and sodium bicarbonate, regarding eGFR decrease by 30% [HR = 1.14 (95% CI: 0.46–2.84), *P* = .77], eGFR decrease by 50% [HR = 0.43 (95% CI: 0.04–4.74), *P* = .50], dialysis [HR = 0.90 (95% CI: 0.12–6.24), *P* = .85], death or prolonged hospitalization [HR = 0.30 (95% CI: 0.03–2.87), *P* = .29] and the combined endpoint occurrence [HR = 0.80 (95% CI: 0.37–1.73), *P* = .57] (Fig. 3; Table 4).

**Table 4:** Proportional hazard risk analysis for secondary outcomes.

	Sodium citrate (N = 62)	Sodium bicarbonate (N = 62)	HR (95% CI)	*P* value
eGFR decrease by 30% (%)	11 (17.7)	8 (12.9)	1.14 (0.46–2.84)	.77
eGFR decrease by 50% (%)	1 (1.6)	2 (3.2)	0.43 (0.04–4.74)	.50
Dialysis (%)	2 (3.2)	2 (3.2)	0.90 (0.12–6.24)	.85
Death/prolonged hospitalization (%)	1 (1.6)	3 (4.8)	0.30 (0.03–2.87)	.29
Combined EP (%)	13 (21)	13 (21)	0.80 (0.37–1.73)	.57

eGFR = estimated glomerular filtration rate, EP = endpoint, IQR = interquartile range, N = number.

The rates of different adverse events of interest were generally similar between the 2 groups (Table 5). Metabolic alkalosis was only observed in the sodium bicarbonate group and gastrointestinal events were higher, but not significant in this group. Drug discontinuation due to adverse events was significantly higher in patients treated with sodium bicarbonate (17.7% vs 4.8%, *P* = .02). In the sodium citrate group drug discontinuation was due to diarrhea (1 patient), epigastric pain (1 patient) and nausea (1 patient). In the sodium bicarbonate group drug discontinuation was due to metabolic alkalosis (4 patients), diarrhea (4 patients), vomiting (1 patient), and epigastric pain (2 patients).

**Table 5:** Safety profile and study drug discontinuation according to treatment group.

	Sodium citrate	Sodium bicarbonate	*P* value
Metabolic alkalosis (%)	0 (0)	4 (6.4)	.11
Nausea (%)	4 (6.4)	7 (11.3)	.52
Bloating (%)	2 (3.2)	5 (8.1)	.43
Vomiting (%)	0 (0)	1 (1.6)	1
Epigastric pain (%)	1 (1.6)	5 (8.1)	.20
Diarrhea (%)	1 (1.6)	4 (6.4)	.17
Medication withdrawal due to AE (%)	3 (4.8)	11 (17.7)	.02

AE = adverse events.

## 4. Discussion

To the best of our knowledge, this is the first randomized controlled trial to compare the effect and safety profile of sodium citrate with those of sodium bicarbonate in patients with CKD and MA. We found that oral sodium citrate and oral sodium bicarbonate had similar effects on kidney function decline; both improved serum bicarbonate levels, but oral sodium bicarbonate was more often associated with treatment withdrawal due to adverse events.

We did not observe a significant difference between the 2 groups in terms of eGFR changes at any time point during the follow-up period. Likewise, a previous randomized trial that evaluated the effect of oral sodium bicarbonate compared to placebo in patients with CKD stages 3 and 4 showed no differences in eGFR rates at any time-point during a period of 24 months.^[8]^ In our study, the eGFR decline in the sodium bicarbonate group was similar to that reported by Ione de Brito-Ashurst et al (−1.88 mL/min/1.73 m^2^).^[9]^ In addition, the eGFR decline in the sodium citrate group after 1 year of treatment was similar to that observed by Phisitkul et al (−1.60 mL/min/1.73 m^2^).^[10]^ In another randomized parallel trial which compared sodium bicarbonate with standard of care in patients with CKD stage 4 and 5, CKD stage 4 patients from the intervention group had a eGFR decreased of −2.30 mL/min/1.73 m^2^, after 12 months of treatment.^[11]^ The rate of eGFR decline in the 2 treatment groups included in our study was slower than in untreated patients or those who received standard of care treatment, from previous studies (−3.79 mL/min/1.73 m^2^ and −5.93 mL/min/1.73 m^2^ respectively).^[9,10]^ Regarding other renal endpoints evaluated in our study, dialysis endpoint in both studied groups was lower than that reported by Ione de Brito-Ashurst et al (6.5%), as well as that reported by Biagio Di Iorio et al (6.9%).^[9,12]^ The potential benefits of sodium bicarbonate on kidney function in patients with CKD have been assessed in a recent systematic review and meta-analysis.^[13]^ Although, seems that sodium bicarbonate may slow CKD progression, the overall evidence is of low certainty, and it is limited by the low number of blinded or placebo-controlled trials. Thus, in interpreting the results and their clinical applicability this aspect should be considered and for stronger conclusions adequately powered randomized trials are required.

Regarding MA correction, we observed a significant increase in serum bicarbonate levels in both groups. These results are in line with those of previous studies, which showed that treatment with either sodium citrate or sodium bicarbonate in the short- and long-term produces a significant improvement in serum bicarbonate levels in patients with metabolic acidosis and CKD stages 3 to 5.^[8–10,12,14]^ However, one caveat is that these studies did not compare the effect of the 2 drugs with each other, but the effect of each drug was compared separately with placebo or standard of care. Our study was not designed to analyze the target dose of each drug for MA correction. We established dosages according to the protocol used in the treatment of MA in our clinic and based on limited data from previous studies.

Current research has shed light on the intricate connection between MA and CKD progression, revealing a compelling pathophysiological link that warrants thorough exploration.^[5,15]^ Mechanisms responsible for GFR reduction include adaptive responses that increase acid excretion but lead to a decline in kidney function.^[15]^ One mechanism consists of intrarenal activation of hormones, such as angiotensin II, aldosterone, and endothelin-1, stimulated by metabolic acidosis, which increases kidney acid excretion, but their chronic upregulation becomes maladaptive and leads to vasoconstriction, inflammation and fibrosis.^[16–18]^ Another mechanism is the stimulation of ammoniogenesis by metabolic acidosis, which, on the one hand, increases acid excretion but, on the other hand, causes complement activation responsible for tubulointerstitial damage and progression of kidney disease.^[19–21]^ Additionally, oxidative stress and reactive oxygen species produced by metabolic acidosis can lead to cellular dysfunction and destruction, further contributing to the progression of kidney disease.^[22]^ Goraya et al showed that treatment with alkali therapy improved metabolic acidosis and was associated with a significant reduction in kidney injury markers (urinary angiotensinogen, urinary neutrophil gelatinase-associated lipocalin, and albuminuria) and preserved kidney function in CKD patients.^[23]^ Thus, it can be claimed that alkali supplementation in the form of sodium citrate or sodium bicarbonate, by determining a significant increase in serum bicarbonate, reduces intrarenal lesional mechanisms and helps slow CKD progression.^[24]^ Notably, in our study, there was a higher use of ACEi/ARBs among patients in the sodium citrate group at baseline. Hypertension is highly prevalent in CKD patients and, according to current guidelines, is treated with one of the 2 classes of drugs. From a mechanistic perspective, blocking angiotensin II may have an antifibrotic effect. Thus, sodium citrate and ACEi/ARBs may produce a beneficial synergistic effect in reducing CKD progression. Although this effect may affect GFR and proteinuria, we did not observe any differences after 12 months of treatment; thus, it cannot be inferred that their combination may influence the endpoints.

Notwithstanding, the rates of different adverse events were statistically similar between the 2 groups, the overall number of patients who stopped treatment due to adverse events was significantly higher in sodium bicarbonate group. This points out that, although the incidence of different adverse events may be comparable, the severity or their impact in terms of tolerability differs between groups. Thus, in interpreting this discrepancy, both the statistical factor and the clinical impact translated into tolerability must be considered. When specifically comparing the rates of various adverse events between the 2 groups, the relatively small number of events may explain the lack of statistical significance, but when the number of adverse reactions leading to drug discontinuation in each group was cumulated, the difference became statistically significant. All 4 cases of metabolic alkalosis were observed in the sodium bicarbonate group, and it led to treatment withdrawal in all cases. Treatment withdrawal due to lack of gastrointestinal tolerability in the citrate group was observed in 3 patients (1 case of diarrhea, 1 case of epigastric pain and 1 case of nausea), while in the bicarbonate group it was observed in 7 patients (4 cases of diarrhea, 2 cases of epigastric pain and 1 case of vomiting). The lower gastrointestinal tolerance of the medication in the sodium bicarbonate group can be explained by the fact that bicarbonate reacts with hydrogen chloride in the gastric lumen, leading to the generation of carbon dioxide gas, which is not the case for citrate. We did not observe any effect on blood pressure or evidence of worsening edema. It’s important to note that the choice between sodium citrate and sodium bicarbonate should be individualized based on the patient’s clinical condition, the underlying cause of acidosis, and other relevant factors such as tolerability. Based on the gastrointestinal tolerance, it could be opined that a rational use of sodium citrate over sodium bicarbonate could be considered in patients with a history of gastrointestinal disorders or those who do not tolerate sodium bicarbonate for correction of MA. An important aspect to consider when prescribing these alkaline supplements is their availability. Sodium citrate could be less or unavailable in some countries compared to sodium bicarbonate for the treatment of MA depending on various factors such as geographical area, local regulations, healthcare infrastructure and local clinical practice.

This study has several strengths, including the number of patients in a single-center study, and that it is the first randomized control trial to compare the effect and safety profile between sodium citrate and sodium bicarbonate. Nevertheless, our study has some limitations, such as the relatively short duration of follow-up, lack of blinding, and single-center nature. Due to the lack of blinding, there could be susceptibility to adverse event reporting bias. Thus, patients who knew that they would receive sodium bicarbonate could report more gastrointestinal side effects. Another limitation could be related to the fact that adherence was measured by self-reporting and not by collecting drug levels or using smart pill bottles. As this was a single-center study, the reproducibility and generalizability of the data could not be applied to all patients, and a randomized, double-blind, multicenter study could resolve this issue. Multicentric, large-scale studies would provide further information regarding the differences between the 2 alkali therapies in terms of benefits and safety for CKD patients with MA.

## 5. Conclusions

In conclusion, oral sodium citrate and oral sodium bicarbonate have a similar effect on kidney function decline, both improve serum bicarbonate level, but oral sodium bicarbonate is associated with higher rates of medication discontinuation due to adverse events in patients with CKD stages 3b-4 and MA.

## Acknowledgments

We are thankful to all participants and staff involved in the study.

## Author contributions

**Conceptualization:** Bogdan Marian Sorohan, Bogdan Obrișcă, Gener Ismail.

**Data curation:** Bogdan Obrișcă, Roxana Jurubiță, Gabriela Lupușoru.

**Formal analysis:** Bogdan Marian Sorohan, Roxana Jurubiță, Gabriela Lupușoru, Camelia Achim.

**Investigation:** Bogdan Marian Sorohan, Roxana Jurubiță, Gabriela Lupușoru, Andreea Andronesi, Georgiana Frățilă, Andreea Berechet, Georgia Micu, Gener Ismail.

**Methodology:** Camelia Achim, Georgiana Frățilă, Andreea Berechet, Georgia Micu, Gener Ismail.

**Resources:** Bogdan Marian Sorohan, Gener Ismail.

**Software:** Bogdan Marian Sorohan, Georgiana Frățilă, Andreea Berechet, Georgia Micu.

**Supervision:** Bogdan Marian Sorohan, Bogdan Obrișcă, Andreea Andronesi, Gener Ismail.

**Validation:** Bogdan Obrișcă, Gabriela Lupușoru, Camelia Achim, Andreea Andronesi, Gener Ismail.

**Visualization:** Bogdan Marian Sorohan, Bogdan Obrișcă, Gabriela Lupușoru, Camelia Achim, Gener Ismail.

**Writing – original draft:** Bogdan Marian Sorohan, Bogdan Obrișcă, Roxana Jurubiță, Gabriela Lupușoru, Camelia Achim, Andreea Andronesi, Georgiana Frățilă, Andreea Berechet, Georgia Micu, Gener Ismail.

**Writing – review & editing:** Bogdan Marian Sorohan, Bogdan Obrișcă, Roxana Jurubiță, Gabriela Lupușoru, Camelia Achim, Andreea Andronesi, Georgiana Frățilă, Andreea Berechet, Georgia Micu, Gener Ismail.
